# The Development and Neuronal Complexity of Bipinnaria Larvae of the Sea Star *Asterias rubens*

**DOI:** 10.1093/icb/icab103

**Published:** 2021-06-28

**Authors:** Hugh F Carter, Jeffrey R Thompson, Maurice R Elphick, Paola Oliveri

**Affiliations:** Department of Genetics, Evolution and Environment, University College London, Darwin Building, Gower Street, London WC1E 6BT, UK; Department of Life Sciences, Natural History Museum, Cromwell Road, South Kensington, London SW7 5BD, UK; Department of Genetics, Evolution and Environment, University College London, Darwin Building, Gower Street, London WC1E 6BT, UK; UCL Centre for Life’s Origins and Evolution (CLOE), University College London, Darwin Building, Gower Street, London WC1E 6BT, UK; School of Biological and Chemical Sciences, Queen Mary University of London, London E1 4NS, UK; Department of Genetics, Evolution and Environment, University College London, Darwin Building, Gower Street, London WC1E 6BT, UK; UCL Centre for Life’s Origins and Evolution (CLOE), University College London, Darwin Building, Gower Street, London WC1E 6BT, UK

## Abstract

Free-swimming planktonic larvae are a key stage in the development of many marine phyla, and studies of these organisms have contributed to our understanding of major genetic and evolutionary processes. Although transitory, these larvae often attain a remarkable degree of tissue complexity, with well-defined musculature and nervous systems. Among the best studied are larvae belonging to the phylum Echinodermata, but with work largely focused on the pluteus larvae of sea urchins (class Echinoidea). The greatest diversity of larval strategies among echinoderms is found in the class Asteroidea (sea stars), organisms that are rapidly emerging as experimental systems for genetic and developmental studies. However, the bipinnaria larvae of sea stars have only been studied in detail in a small number of species and although they have been relatively well described neuro-anatomically, they are poorly understood neurochemically. Here, we have analyzed embryonic development and bipinnaria larval anatomy in the common North Atlantic sea star *Asterias rubens*, using a variety of staining methods in combination with confocal microscopy. Importantly, the chemical complexity of the nervous system of bipinnaria larvae was revealed through use of a diverse set of antibodies, with identification of at least three centers of differing neurochemical signature within the previously described nervous system: the anterior apical organ, oral region, and ciliary bands. Furthermore, the anatomy of the musculature and sites of cell division in bipinnaria larvae was analyzed. Comparisons of developmental progression and molecular anatomy across the Echinodermata provided a basis for hypotheses on the shared evolutionary and developmental processes that have shaped this group of animals. We conclude that bipinnaria larvae appear to be remarkably conserved across ∼200 million years of evolutionary time and may represent a strong evolutionary and/or developmental constraint on species utilizing this larval strategy.

## Introduction

Species from many marine phyla develop via a biphasic lifestyle, transitioning from a free-swimming planktonic larva to a more sedentary benthonic adult. Although transitory, the larval phase often develops a remarkable degree of tissue complexity, including a nervous system and musculature. The study of the planktonic larvae of several marine taxa has been important for our understanding of species dispersal (Scheltema 1986) and developmental processes (Raff 2008). Furthermore, since the majority of animal phyla are marine, comparative studies of genetic networks that direct the molecular and cellular processes of larval development are key to our understanding of the evolutionary processes that have shaped the animal kingdom (Davidson et al. 2002; Davidson and Erwin 2006; Peter and Davidson 2015; Dylus et al. 2016).

Of the phyla with planktonic larvae, the Echinodermata is perhaps the best understood, having been used as experimental organisms for studies of early development for over a century (Boveri 1893). Unlike their pentaradial adults, echinoderm larvae are bilaterally symmetrical, although they differ widely in morphology between and within classes (McEdward and Miner 2001; Young et al. 2002). Echinoderm larvae are typically characterized based upon how nutrition is obtained before metamorphosis: either free-feeding planktotrophs with complex ciliary bands, or lecithotrophs dependent on a maternally derived yolky substance (Zamora et al. 2020). Within these categories, there is considerable diversity and some species display highly derived developmental progressions that do not fall neatly within these two sub-divisions (McEdward 1995; Byrne et al. 2007). Although indirect-developing echinoderm larvae vary extensively in morphology within and across classes (Wray 1992; Hart et al. 1997; McEdward and Miner 2001; Byrne and Selvakumaraswamy 2002; Young et al. 2002), phylogenetic comparative studies strongly suggest that indirect development, through a free-swimming and feeding planktotrophic larva, represents the ancestral strategy for all echinoderm*s* (McEdward and Janies 1997; Peterson et al. 2000; Raff and Byrne 2006). To understand conserved and divergent morphologies, tissues, and cell types across echinoderm larvae, a better understanding of their molecular signatures in indirect-developing, planktonic larvae is crucial.

Among echinoderms, the Asteroidea or sea stars have the greatest variety of described larval strategies (McEdward and Miner 2001) and are emerging as experimental systems for developmental and genetic studies (Stewart et al. 2015; Byrne et al. 2020; Cary et al. 2020). The bat-star *Patiria miniata* in particular has become an important comparative resource for understanding divergence and conservation of gene regulatory network architecture over long evolutionary timescales (Cary and Hinman 2017). Additionally, a growing number of asteroid species now have well-annotated genome assemblies, making this a particularly pertinent moment for a detailed assessment of asteroid larval development (Hall et al. 2017; Cary et al. 2018; Ruiz‐Ramos et al. 2020).

The bipinnaria, a free-swimming planktotrophic larva, is the most phylogenetically widespread larval form among asteroids and is considered to represent the ancestral larval form of the class (McEdward 1995; Raff and Byrne 2006). In most cases, the bipinnaria is followed by a more complex brachiolaria, from which arises an attachment complex before metamorphosis (Haesaerts et al. 2005; Murabe et al. 2008). The bipinnaria is characterized by two bilaterally symmetrical ciliary bands and an open, functional gut. Although superficially simple, they require a surprising degree of neuronal complexity, both for environmental sensing and for coordination of the ciliary bands, which play a dual role in feeding and locomotion (Burke 1983; Lacalli et al. 1990; Hinman and Burke 2018). Despite its key evolutionary position, the bipinnaria larva has only been the subject of detailed investigation in a small number of species and although the extent of the nervous system has been thoroughly described in both this stage and the later brachiolaria, there remains only a rudimentary understanding of the molecular complexity of the asteroid larval nervous system (Moss et al. 1994; Byrne and Cisternas 2002; Murabe et al. 2008).

One of the most abundant northern hemisphere asteroids, *Asterias rubens*, populates the Atlantic Ocean from northern Norway to Senegal in the east and northern Canada to North Carolina in the west at depths of 0–650 m (Budd 2008). *Asterias rubens* undergoes the common larval transition of bipinnaria to brachiolaria, but its early larval development from the first cleavage to free-swimming bipinnaria has not been studied in detail for more than a century (Gemmill 1914). While studies of larval development are lacking, adult *A. rubens* has been an experimental system for functional characterization of neuropeptide signaling for several decades and a large set of taxon-specific antibodies against multiple neuropeptides has been developed (Elphick et al. 1995; Odekunle et al. 2019; Zhang et al. 2020). This has been facilitated by the rapidly increasing availability of transcriptomic and genomic resources for this species (Semmens et al. 2016). The expression patterns of many neuropeptides have been described in adults (Newman et al. 1995; Lin et al. 2017; Tian et al. 2017; Cai et al. 2018) and the brachiolaria stage (Mayorova et al. 2016), allowing for comparison of conserved or differential molecular signatures between the larval and adult body plans. *Asterias rubens* is also phylogenetically well-suited for comparative studies of the larval nervous system. It belongs to the order Forcipulatida and is thus distantly related to the valvatids *P. miniata* and *P. pectinifera*, but closely related to another forcupilatid—*Pisaster ochraeus* (Mah and Foltz 2011). These species are the asteroids in which the larval nervous system has previously been characterized in detail, and studies on *A. rubens* thus allow for comparisons across both deep and shallow phylogenetic distances.

To precisely understand the development of bipinnaria larvae from a molecular and cellular perspective, and to facilitate evolutionary comparisons across echinoderms and other marine invertebrates, we have surveyed the early and larval development of *A. rubens* by analyzing the expression of selected proteins and cell proliferation. In particular, we have focused on the larval nervous system of the 2-week-old bipinnaria, a point after the main feeding and locomotory structures have developed, but before the development of additional brachiolaria structures, using a combination of antibodies, some of which are here uniquely applied to echinoderm larvae. This work enables comparisons of the development of the nervous system and other structures across the planktonic larvae of the Echinodermata to better provide an understanding of the evolutionary and developmental processes that have shaped the biodiversity of the group.

## Materials and methods

### Gamete collection and embryo culture

Gamete collection and embryo culture followed Jarvela and Hinman (2014). Following fertilization, eggs were incubated at either 12°C or 15°C, and the embryonic development is allowed to proceed (see Supplementary Methods). Developmental progression was checked hourly until embryos had undergone their fifth cleavage, then at least once daily until the culture reached the bipinnaria stage. At each time point, several individuals (>30) were mounted on glass slides and visualized using light microscopy. Embryos and larvae were collected at different stages for further processing.

### Immunostaining and cell proliferation

Immunostaining and assays of cell proliferation followed procedures reported by Thompson et al. (2021). For each developmental time point of interest, 4,000–7,000 embryos or larvae were fixed in 4% PFA, permeabilized in ice-cold methanol, and stored in blocking buffer (BB). Samples were incubated in primary antibodies at 37°C for 1.5 h, then incubated in secondary antibodies for 1 h at room temperature (RT) before final incubation with DAPI for 5 min at RT. For antibody combinations and further details, see Supplementary Materials. Dividing cell nuclei were labeled using the Click-iT^®^ EdU Alexa Flour^®^ 555HCS kit (Life Technologies) according to the manufacturer’s protocol.

### Microscopy

Differential interference contrast (DIC) and epi-fluorescent images were produced using a Zeiss AxioImager M1 microscope and Zeiss AxioCamHRc camera. Confocal microscopy was performed using a LSM 800 confocal microscope or Leica SPEinv inverted confocal microscope. Image processing and analysis were carried out using ImageJ version 2.0.0.0.

## Results

### *Asterias rubens* develops canonically to a bipinnaria larva

**Fig. 1.**
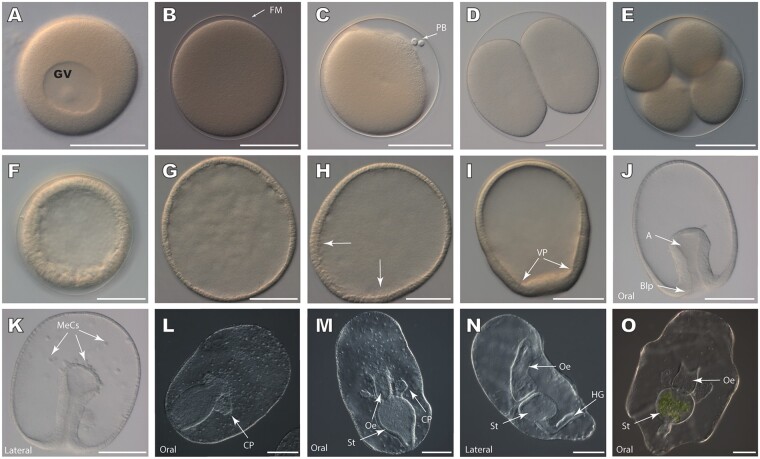
Developmental progression of *A. rubens* from fertilized egg to bipinnaria larva. (**A**) Unfertilized immature oocyte with the germinal vesicle. (**B**) Fertilized oocyte without germinal vesicle. Note the presence of fertilization membrane. (**C**) Fertilized oocyte with polar bodies. (**D**) Two-cell stage embryo following the first cleavage. (**E**) Four-cell stage embryo. Cleavages are roughly equal, giving rise to blastomeres of roughly equal size within the fertilization membrane. (**F**) Blastula-stage embryo, consisting of approximately 250 cells. Embryos are round at this stage, due to constraints imposed by the fertilization membrane. (**G**) Blastula-stage embryo following the loss of the fertilization membrane. (**H**) Swimming blastula-stage embryo. (**I**) Blastula-stage embryo. Elongation has taken place along the anteroposterior axis, and thickening and flattening of the vegetal plate is evident along the vegetal end of the embryo. (**J**) Early gastrula-stage embryo. Archenteron has invaginated and has thinner walls at the blind end than in the rest of the tube. (**K**) Mid-gastrula stage embryo. Archenteron has thickened relative to early gastrula-stage embryos seen in (J). Additionally, many mesenchymal cells have separated from the tip of the archenteron. (**L**) The lumen of archenteron has expanded and two coelomic pouches have formed in the developing gut. (**M**) Immature bipinnaria stage larva. The gut has differentiated into the esophagus, stomach, and intestine, and coelomic pouches are well-developed. Numerous mesenchymal cells are present. (**N**) Lateral view of bipinnaria larva. A well-developed, tripartite gut is present, as well as the hydropore canal, which connects one of the coelomic pouches to the exterior of the animal. (**O**) Bipinnaria larvae with tripartite gut and two elongate coelomic pouches. Green algae are present in the stomach of the animal, showing that it has started feeding. GV, germinal vesicle; PB, polar bodies; FM, fertilization membrane; VP, vegetal plate; A, archenteron; MeCs, mesenchymal cells; CP, coelomic pouch; St, stomach; Oe, esophagus, HG, hind gut. Scale bars are 75 µm.

To better understand the tempo and mode of *A. rubens* development, we optimized culture conditions to produce thousands of synchronously developing embryos. Development was followed at two different temperatures (12°C and 15°C), corresponding to the UK coastal temperature range during the spawning season, in three independent batches to produce a staging system (Fig. 1 and Supplementary Fig. S1). A full account of larval development can be found in the Supplementary Material. Briefly, maturation of oocytes was induced with 1-methyl-adenine leading to loss of the germinal vesicle (Fig. 1A and B). The fertilization membrane elevates rapidly after sperm addition and the first cleavage is complete by 2 hpf at 15°C and 3 hpf at 12°C (Fig. 1D). In each batch, subsequent cleavages are holoblastic and equal for roughly 9–11 cycles, occurring on average every 2 h at 15°C and 3 h at 12°C. The fertilization membrane constrains growth until ∼24 hpf (Fig. 1G) with its loss accompanied by a pronounced elongation along the animal/vegetal axis and a thickening of cells in the vegetal half of the embryo (Fig. 1H). The vegetal plate flattens and thickens before gastrulation (Fig. 1I), producing an invaginating archenteron that reaches half the length of the embryo by 46 hpf. The lumen of the archenteron gradually decreases in diameter as the walls thicken and the archenteron extends in length (Fig. 1J and K). By 70 hpf, mesenchyme cells arising from the tip of the archenteron are apparent in the blastocoel (Fig. 1K) and these bud-off into the blastocoel throughout gastrulation (Fig. 1K–M). By 92 hpf, two differently sized coelomic pouches are apparent and the central part of the archenteron expands to form the proto-stomach (Fig. 1L). The immature bipinnaria (122 hpf) has a tripartite gut with an esophagus, stomach and hind-gut (Fig. 1M and N), and mesenchymal cells that have ingressed into the blastocoel start to show considerable morphological differentiation (Supplementary Fig. S3).

In the early bipinnaria larva (Fig. 1N), the developing esophagus bends toward the oral surface and fuses with a pronounced depression, the oral cavity. The lower intestine bends orally and forms the anus. The anus is located on the post-oral lobe (Fig. 1N), one of the two prominent lobes on the oral surface of the larva. The other lobe, the pre-oral lobe, overhangs the mouth and forms the oral hood (Fig. 1N). These lobes are encircled by the pre-oral and post-oral ciliary bands, dense accumulations of ciliated cells. The pre-oral band encircles the anterior end of the larva and the oral hood, while the post-oral ciliary band surrounds the posterior portion of the larva and connects to the lower lip of the oral cavity (Fig. 1O).

### Bipinnaria larvae have two distinct populations of muscle cells

**Fig. 2.**
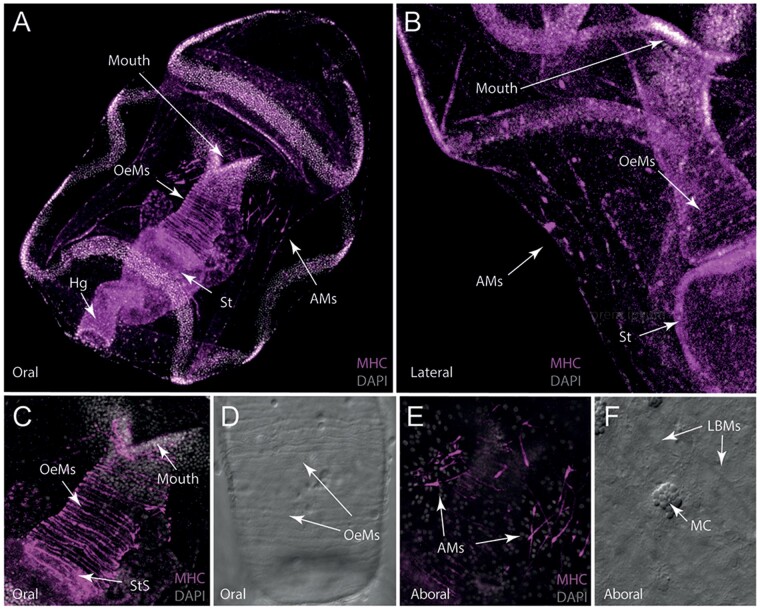
Musculature of bipinnaria larva of *A. rubens*. (**A**) Maximal projection confocal image showing the oral view of myosin heavy chain (MHC, false colored in magenta) immunoreactivity and cell nuclei visualized with DAPI (gray) in a 2-week-old larva. Strong MHC immunostaining can be seen in three populations of muscle cells: fibrous longitudinal muscles encircling the esophagus, and two clusters of aboral muscles associated with the dorsal surface. (**B**) Lateral view of maximum projection confocal image showing a high-magnification view of MHC-immunoreactive aboral muscles located on the dorsal surface of the larva, as well as the esophageal muscles. (**C**) Oral view of maximum projection confocal image showing a high-magnification view of MHC-immunoreactive muscles surrounding the esophagus. (**D**) Bright-field (with DIC) image showing the esophageal muscles surrounding the stomach, which exhibit MHC-immunoreactivity as shown in (C). (**E**) High-magnification image showing MHC-immunoreactivity in aboral muscle cells. These cells are arranged into two clusters on the dorsal surface of the animal and are thicker at one end with a tapering morphology at the other end. (**F**) Bright-field (with DIC) image of aboral muscle cells and their proximity to mucous cells embedded in the ectoderm of the larva. OeMs, esophageal muscles; AMs, aboral muscles; St, stomach, Sts, stomach sphincter; MC, mucous cell; Hg, hind gut.

To investigate the molecular basis of the development of larval structures and identify cell types, molecular markers were used to characterize tissues and cells in *A. rubens* larvae at different developmental time points. Musculature was visualized using an antibody against Myosin Heavy Chain (Fig. 2A), which has been shown to label fore-gut muscles in sea urchin larvae (Annunziata et al. 2014). In bipinnaria, MHC-immunoreactivity is present in two populations of cells: the muscles encircling the esophagus (Fig. 2C and D) and bilateral clusters of six to eight cells near the aboral surface (Fig. 2B, E, and F). Esophageal muscle cells are elongate with a prominent nucleus but do not individually encircle the esophagus (Fig. 2D). These cells produce a strong peristaltic contraction, moving food particles through the digestive system (Supplementary Fig. S4A and B). Immunoreactivity is strongest in cells forming the esophageal–stomach sphincter (Fig. 2C). Aboral muscle cells have similar elongate morphologies to esophageal muscles (Fig. 2E and F) but form a clearly distinct structure capable of producing contractions in the aboral surface (Supplementary Fig. S4C and D).

### Bipinnaria larvae have an extensive nervous system

**Fig. 3.**
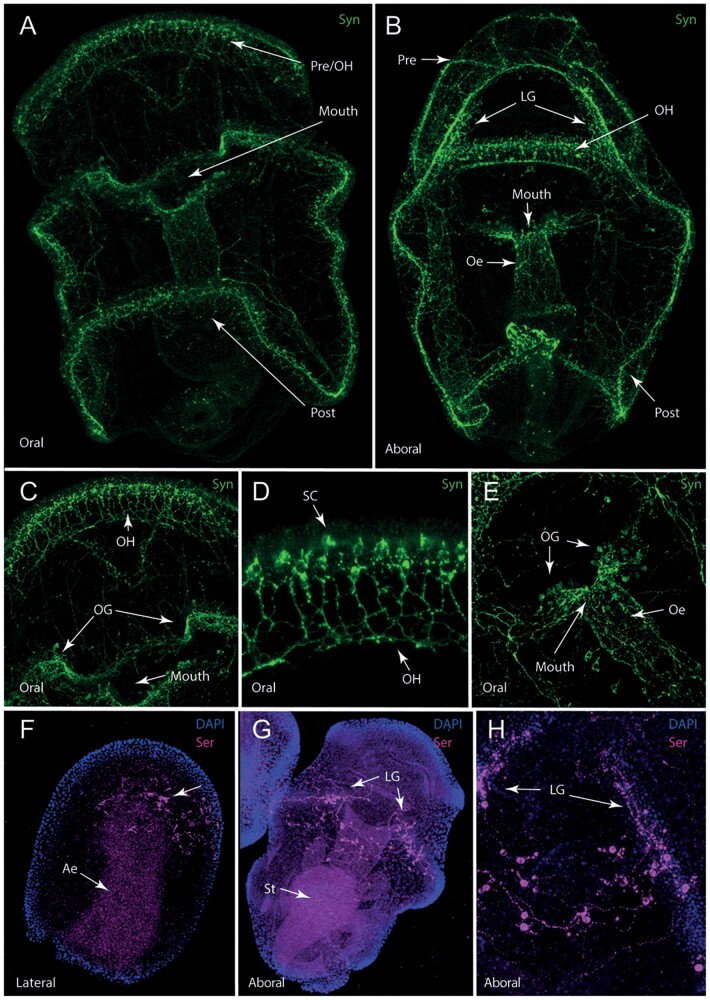
Architecture and morphology of the nervous system in *A. rubens* embryos and larvae*.* (**A**) Maximum projection in oral view showing synaptotagmin-immunoreactivity revealed by 1E11 antibodies (green) in the *A. rubens* bipinnaria larva. Strong immunoreactivity is present in the ciliary bands around the mouth and along the gut. In addition to these major areas of labeling, synaptotagmin-immunoreactive projections are also present in other regions of the larva. (**B**) Synaptotagmin-immunoreactivity in a maximum projection ventral view of a bipinnaria larva. Strong staining is present in the pre-oral ciliary band and the oral hood, as well as in the lateral ganglia and post-oral ciliary band. Innervation can be seen lining the esophagus as well as around the mouth. (**C**) High-magnification maximum projection image of synaptotagmin-immunoreactive neurons in the oral hood with orally directed projections. Additionally, labeling of clustered neurons of the oral ganglia can be seen on either side of the mouth. (**D**) High-magnification maximum projection image of synaptotagmin-immunoreactive neurons of the oral hood shown in (C), which can be interpreted as sensory cells with downward projecting processes. (**E**) High-magnification maximum projection image of synaptotagmin-immunoreactive neuronal cell bodies of the oral ganglia proximal to the mouth and with immunostained processes distributed along the length of the esophagus. (**F**) Gastrula stage maximum projection embryo stained with serotonin antibodies (magenta) and with cell nuclei labeled using DAPI (blue). Immunoreactivity is strongest in numerous ectodermal cells, interpreted to be neuronal precursors. Immunostaining in the archenteron is interpreted as background staining. (**G**) Maximum projection ventral view of an immature bipinnaria showing serotonin immunoreactive cells and processes along the ventral surface. These cells are interpreted to be serotonergic neurons and are arranged into 2 clusters in the lateral ganglia. Immunostaining in the stomach is interpreted as background staining. (F) High-magnification maximum projection image of serotonin immunoreactive cells in the lateral ganglia, showing stained cell bodies and neuronal processes. Abbreviations: Pre, pre-oral ciliary band; OH, oral hood; Post, post-oral ciliary band; LG, lateral ganglia; Oe, esophagus; OG, oral ganglia; SC, sensory cells.

We next aimed to characterize the *A. rubens* bipinnaria nervous system and how it innervates both the musculature and remainder of the larva. The full extent of the larval nervous system was visualized using the pan-neuronal marker 1E11, which labels the synaptic vesicle trafficking protein synaptotagmin (Fig. 3A–E; Nakajima et al. 2004). Synaptotagmin immunoreactivity in cells of the larval nervous system is concentrated in the ciliary bands, oral hood and mouth, with projections forming a meshwork around the esophagus and extending loosely across the rest of the body (Fig. 3A–E). Each ciliary band is innervated with a prominent central fiber surrounded by synaptotagmin-immunoreactive neuronal cells laterally positioned on each side (Supplementary Fig. S5A). This central fiber seems to be formed from a bundle of nerve fibers and is least organized where it passes over the oral surface of the post-oral lobe (Fig. 3A). Numerous neurites of variable length project laterally from the ciliary band, primarily toward the oral surface (Supplementary Fig. S5B).

A population of neurons in the oral hood has short apical processes (Fig. 3D) and may correspond with a well-characterized population of sensory neurons in the apical organ of other echinoderms (Byrne et al. 2007). Synaptotagmin-immunoreactive neurites and neuronal projections extend orally from the basal surface of these cells toward the oral pit, forming a prominent band around its anterior (Fig. 3C and D). On either side of the mouth are two prominent ganglia, from which projections connect to both the post-oral ciliary band nerve and form a network surrounding the esophagus (Fig. 3C and E). Gut innervation is concentrated around the esophagus with limited immunoreactivity in the mid- and hindgut, although some synaptotagmin-immunoreactive cells were also identified around the anus (Fig. 3A). Outside of the ciliary bands and feeding apparatus, the only neuronal cell bodies observed were a loose cluster of cells spanning the mid-aboral surface (Supplementary Fig. S6).

### Neuronal complexity of *A. rubens* bipinnaria larvae

In other echinoderm larvae, multiple neuronal types have been identified using markers for neurotransmitters, neuropeptides, or specific transcription factors (Burke et al. 2006; Slota and McClay 2018; Wood et al. 2018). We have used several antibodies raised against these markers to characterize the bipinnaria nervous system of *A. rubens*.

In echinoderms, the neurotransmitter serotonin is present in a subset of apical organ neurons, anterior ganglia considered to be the central nervous system of larvae (Byrne et al. 2007). We used an anti-serotonin antibody to investigate the development and extent of the serotonergic nervous system in *A. rubens* (Fig. 3F–H and Supplementary Fig. S6). Serotonin-immunoreactive cells were first observed at the gastrula stage (∼46 hpf) as a scattered cluster on one side of the animal half of the embryo (Fig. 3F and Supplementary Fig. S7). In 1-week-old larvae, serotonergic neurons form two clusters at the anterior end of the developing post-oral ciliary band. These develop into the lateral ganglia on the aboral surface of 2-week-old larvae (Fig. 3G and Supplementary Fig. S7C). These ganglia are connected across the aboral surface by a loose network of cells and projections (Fig. 3H). We found no evidence of serotonin-immunoreactivity on the oral surface or in the oral region at any stage analyzed.

**Fig. 4.**
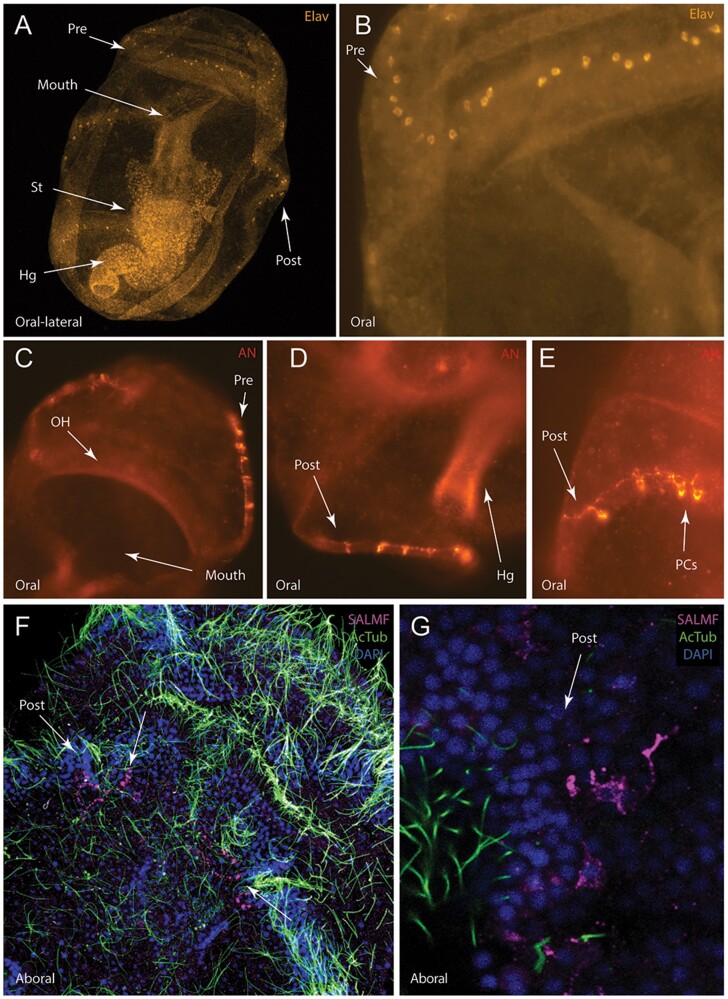
Neuronal subpopulations expressing ELAV or neuropeptides (AN-peptide, SALMFamide-S2) in *A. rubens* larvae. (**A**) Maximum projection confocal image of the oral view of the *A. rubens* bipinnaria showing ELAV-immunoreactivity (orange). Immunostaining is strongest in distinct subpopulations of cells within the pre-oral and post-oral ciliary bands. (**B**) High-magnification confocal image in a maximum projection of ELAV-immunoreactive cells in the pre-oral ciliary band and oral hood. Note that neuronal processes are not labeled by the ELAV-antibodies and immunoreactivity is present in some, but not all, ciliary band neurons. (**C**) High-magnification light microscope image of the oral hood and pre-oral ciliary band showing AN-peptide immunoreactive neuronal cells and processes (red). (**D**) High-magnification maximum projection light microscope image of the post-oral ciliary band showing AN-peptide immunoreactive neuronal cells and processes. (**E**) High-magnification maximum projection light microscope image of the post-oral ciliary band showing AN-peptide immunoreactive neuronal cells and processes. (**F**) Maximum projection confocal image of SALMFamide-S2-immunoreactive processes (pink) in the anterior loop of the post-oral ciliary band and in projections extending onto the oral surface in a larva that has also been labeled for acetylated tubulin (green) and with a nuclear marker (DAPI; blue). (**G**) High-magnification image showing SALMFamide-S2-immunoreactive cells and their processes in the post-oral ciliary band. Abbreviations: Pre; pre-oral ciliary band; Post, post-oral ciliary band; St, stomach; Hg, hindgut; OH, oral hood; PCs, pyramidal cells.

Previous studies have identified sub-populations of neurons that express genes encoding different neuropeptide precursors in sea urchin larvae (Wood et al. 2018). To further characterize neuronal cell types in the ciliary band nervous system of *A. rubens*, we used antibodies raised against the RNA-binding protein ELAV (Garner et al. 2016), considered a specific neuronal marker, a sea urchin AN-peptide-type neuropeptide (Perillo et al. 2018) and the *A. rubens* SALMFamide neuropeptide (S2) (Yun and Thorndyke 2012). ELAV-immunoreactivity was observed in cell bodies of some, but not all ciliary band neurons of 2-week-old larvae (compare Figs. 3A and 4A). Immunoreactivity was also observed in the lower gut and stomach (Fig. 4A and B). Conversely, AN-peptide antibodies label four sets of four to five pyramidal cells in the lateral portions of both ciliary bands. The cells of each set are connected by lateral neuronal projections in the ciliary bands (Fig. 4C–E). Two sets of cells are paired on either side of the pre-oral ciliary band running oral-aborally (Fig. 4C) and the remaining cells are paired on either side of the post-oral ciliary band beside the post-oral lobe (Fig. 4D and E). Antibodies against SALMFamide-S2 label a small bilaterally arranged set of ciliary band neurons in the anterior loop of the post-oral ciliary band (Fig. 4F and G). Short S2-immunoreactive projections extend posteriorly from the ciliary bands across the aboral surface. Importantly, these neurons are located anteriorly to the lateral ganglia and appear distinct from the adjacent serotonin-immunoreactive cells. Collectively, these data suggest a degree of sub-functionalization within the ciliary band.

**Fig. 5.**
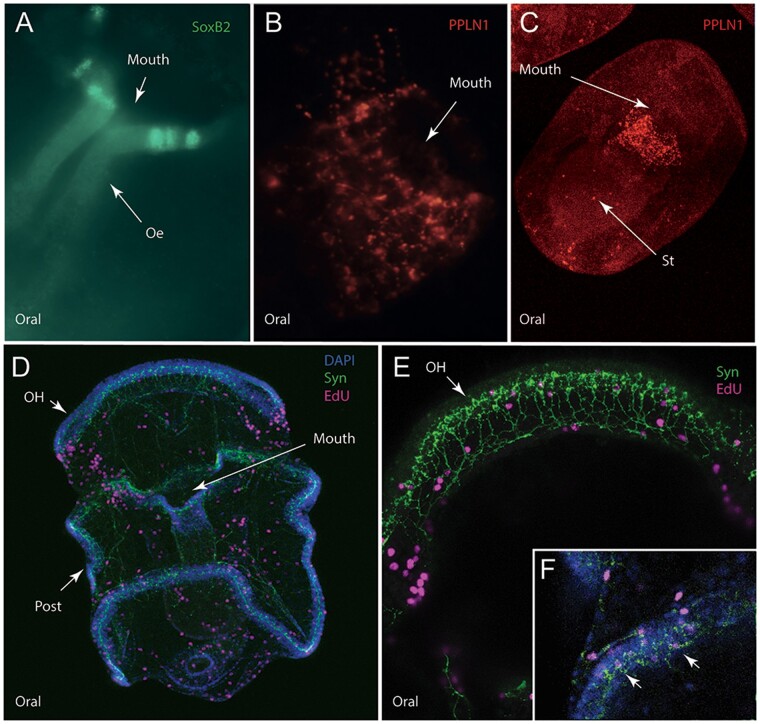
Neuronal subpopulations expressing SoxB2 or the neuropeptide ArPPLN1b and cells undergoing proliferation in *A. rubens* larvae. (**A**) Light microscope image showing immunostaining of a sub-population of cells expressing the transcription factor SoxB2 (green) in the mouth region of a bipinnaria larva. (**B**) Antibodies to the neuropeptide ArPPLN1b (red) reveal immunostained cells and processes around the mouth of a bipinnaria larva in a maximum projection confocal image. (**C**) As in bipinnaria larvae, in immature bipinnaria, ArPPLN1b -immunoreactivity is strongest in a subpopulation of cells around the mouth. (**D**) Cell proliferation in a bipinnaria larva labeled using the marker EdU (magenta) together with immunostaining for synaptotagmin using 1E11 antibodies (green) and DAPI (blue). EdU-positive cells are located throughout the larva but are most strongly concentrated in the ciliary bands. Cell proliferation is most evident along the edges of the pre-oral lobe and the edges of the mouth. Maximum projection of the confocal image. (**E**) Enlargement of D showing the oral hood (arrow). (**F**) Projection of a single confocal slide of a portion of the ciliary band showing an EdU-positive nucleus adjacent to the 1E11 staining. Abbreviations: Oe, esophagus; St, stomach; OH, oral hood; Post, post-oral ciliary band.

Antibodies against the *A. rubens* pedal peptide-type neuropeptide ArPPLN1b (Lin et al. 2017) and SoxB2, a transcription factor expressed during nervous system development (Garner et al. 2016), labeled a sub-population of neurons in the oral region of the bipinnaria. An ArPPLN1b-immunoreactive cluster of cells with associated projections surrounds the mouth and anterior end of the esophagus (Fig. 5B and C) and resemble synaptotagmin-immunoreactive oral neurons.

ArPPLN1b-immunoreactive cells are first observed around the newly formed esophagus of 1-week-old larvae (Supplementary Fig. S8) as a loose network of cells and become prominently localized in projections encircling the entrance to the esophagus by 2 weeks post-fertilization (Fig. 5C). Conversely, SoxB2 was observed only in three small, discrete clusters of cells around the mouth, just above the entrance to the esophagus (Fig. 5A). These cells are morphologically distinct from ArPPLN1b-immunoreactive cells, consistent with different roles for the molecular markers; that is, SoxB2 is involved in neuronal cell precursor specification whereas ArPPLN1b is likely functional in differentiated neurons (Cunningham et al. 2008).

In summary, these data reveal a remarkable diversity of neuronal cell types, highlighting the complexity of the larval nervous system in *A. rubens*.

### Cell proliferation in larval growth and development

As larval growth proceeds, the bipinnaria nervous system of *A. rubens* increases in complexity and the ciliary bands become more defined. Therefore, we investigated the role of cell proliferation in the development of the larval nervous system, with particular focus on the growth of the ciliary bands and associated innervation.

To identify sites of genetic replication as a precursor to cell division during growth, we used 5-ethynyl-2′-deoxyuridine (EdU), a nucleoside analog of thymidine that is incorporated into newly synthesized DNA and thus marks cells in the S-phase (Zeng et al. 2010). In the blastula and early gastrula, no specific patterns were observed, with EdU marked S-phase cells present throughout the embryo (Supplementary Fig. S9). Two-week-old bipinnaria show continued cell progression through S-phase at reduced levels throughout the embryo with EdU marked cells concentrated in the ciliary bands (Fig. 5D and E). Within the ciliary bands, these cells are most apparent on either side of the pre-oral lobe and the edges of the oral pit (Fig. 5D). EdU marked cells are also prominent in the coelomic pouches.

To better understand where cells in S-phase in the ciliary band occurred relative to the tissues of the nervous system, EdU marked samples were co-stained with 1E11. Generally, co-localization was absent, consistent with the notion that terminally differentiated neurons do not re-enter the cell cycle (Jukam and Desplan 2010). However, a small subset of cells appears to have both nuclear EdU and cytoplasmic synaptotagmin-immunoreactivity (Fig. 5F andSupplementary Fig. S9D–G), and these could be interpreted as either neurons in S-phase or as polyploid cells.

## Discussion

### Conservation of the bipinnaria body plan

Our findings (Fig. 1) reveal that the general body plan and early developmental progression of the bipinnaria larvae of *A. rubens* are similar to that of previously studied asteroids (Hyman 1955; Nakajima et al. 2004; Dyachuk and Odintsova 2013; Pernet et al. 2017). This conservation of development and structure is remarkable given the considerable phylogenetic divergence between studied asteroid species (Supplementary Fig. S10). *Patiria miniata* and *A. rubens* have nearly identical bipinnaria larvae despite representing different asteroid super-orders that diverged ∼200 million years ago (Lafay et al. 1995; Linchangco et al. 2017). The only notable variation between species is the speed of larval development. The feeding bipinnaria is present by 3 dpf in *P. miniata* and *P. pectinifera* (Murabe et al. 2021) but not until 5–6 dpf in *Pisaster ochraceus* and *A. rubens* (Pia et al. 2012). Our work also shows more rapid developmental rates in larvae reared at higher temperatures (Supplementary Fig. S1), supporting the view that temperature, not inherent developmental regulation, is the main factor determining the speed of larval development (Cataldo et al. 2005).

In indirect developing asteroids, morphological deviations from the bipinnaria body plan are only apparent at relatively late stages in larval development, such as in members of the genus *Luidia* (Wilson 1978), or in post-metamorphic juveniles (Morris 2009; Pernet et al. 2017). Such limited variation in early bipinnaria development suggests a strong evolutionary or developmental constraint on this stage for species utilizing this larval strategy. Our work, and the ubiquity of the bipinnaria across asteroid phylogeny, further supports the hypothesis that an indirect developing, feeding, and free-swimming larva represents the ancestral mode of development for sea stars (Raff and Byrne 2006). We also note that despite the diversity of non-bipinnaria asteroid larvae, none retain bipinnaria derived structures and there appear to be no intermediate forms (McEdward and Janies 1997). We therefore hypothesize the presence of a developmental switch between two stable larval strategies (planktotrophic bipinnaria and largely direct developing lecithotrophs) rather than gradual morphological shifts.

### Neuronal complexity in the early larvae

**Fig. 6.**
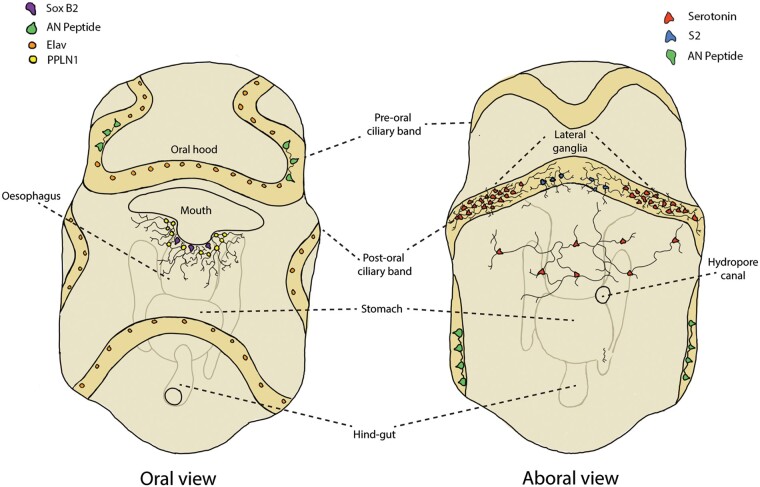
Diagrammatic representation of neuronal subpopulations in *A. rubens* bipinnaria larva. (**Left**) Oral view showing subpopulations of neurons expressing ELAV, SoxB2, and the neuropeptides AN-peptide and ArPPLN1b. The mouth appears to be a center of neuronal complexity, with at least two distinct subpopulations of neurons present. (**Right**) Aboral view showing subpopulations of serotonergic neurons and neurons expressing neuropeptides (SALMFamide-S2 and AN peptide).

Our results show that just 2 weeks post-fertilization, the bipinnaria has developed a complex nervous system extending throughout the body with distinct central and peripheral components. The central nervous system consists of the concentration of neuronal cell bodies in the anterior apical region, while the peripheral nervous system comprises the neurons along with the ciliary bands and around the mouth as well as projections innervating the rest of the body (Fig. 6). This complexity is consistent with previous studies of the bipinnaria nervous system (Nakajima et al. 2004) and subdivisions into central and peripheral components are a shared organizational feature of the nervous systems of echinoids and asteroids (Hinman and Burke 2018). This study is the first, however, to show the scale of neuronal diversity in the early bipinnaria. Within the centralized nervous system, we have identified at least three centers of neurochemical complexity: the anterior end of the post-oral ciliary band around the apical organ, the cells of the oral opening, and additional neurons spread along with the remainder of the ciliary bands (Fig. 6). These correspond with the main concentrations of neurons previously identified in the bipinnaria (Chee and Byrne 1999; Hinman and Burke 2018). Since the primary role of planktonic larvae is to find and consume food, neuronal complexity within structures involved in feeding and locomotion suggests that the majority of these neurons play a role in the coordination of these processes.

In the oral region, our results show that the neuropeptide ArPPLN1b is expressed in a loose network of neurons surrounding the anterior portion of the esophagus (Fig. 5B). While the expression pattern of this neuropeptide has not been described previously in larvae, studies in adult *A. rubens* have revealed that it causes muscle relaxation and so is also known as starfish myorelaxant peptide (Kim et al. 2016; Lin et al. 2017). The proximity of ArPPLN1b-immunoreactivity to the larval esophageal muscles suggests a potentially conserved role in neuromuscular signaling between larvae and adults, despite the full breakdown and reassembly of the nervous system during metamorphosis. Detailed neuroanatomical work will, however, be required to determine if interactions exist between these neurons and esophageal muscles.

We also identified expression of the neurogenic transcription factor SoxB2 in the oral region, distinct from ArPPLN1b-immunoreactive neurons around the opening of the esophagus (Fig. 5A). SoxB2 is associated with the nervous system throughout the animal kingdom (Royo et al. 2011) and is specifically involved in neurogenesis in the echinoid pleuteus larvae foregut, where it is the first marker expressed in neuronal precursors (Garner et al. 2016). Oral neurons develop *in situ* in echinoid larvae (Wei et al. 2011) and we hypothesize the presence of a similar system in asteroids, with SoxB2 expressed in neuronal precursors and ArPPLN1b expressed in differentiated oral/esophageal neurons.

The RNA binding protein ELAV is expressed in a subset of cells along the length of both ciliary bands, in agreement with previous studies of echinoid and asteroid larvae (Yankura et al. 2013; Garner et al. 2016). Unlike these studies, we found no evidence of ELAV-immunoreactive cells in the oral region despite the extensive neuronal array labeled by synaptotagmin antibodies. This suggests a limitation of ELAV as a consistent marker of all major neuronal fields, at least in *A. rubens* larvae.

AN-peptide-immunoreactivity is localized in a unique pattern of two paired clusters of cells in the pre- and post-oral ciliary bands (Fig. 4C–E and Supplementary Fig. S8A), distinct from all other neuronal markers in this study. This pattern is also distinct from the expression pattern observed in larvae of *S. purpuratus* (Perillo et al. 2018), where AN peptide antibodies label lateral ganglia and apical organ neurons that co-express serotonin. That the expression pattern of this neuropeptide is so different between asteroids and echinoids suggests that it may have evolved distinct functions in each class. Therefore, further investigation of AN peptide expression and function in echinoderm larvae may provide valuable insights into the neuronal complexity and evolutionary history of early bipinnaria larvae.

### Serotonergic nervous system and the apical organ

The serotonergic nervous system has been extensively studied across the larvae of the Echinodermata, although research has primarily focused on echinoids and asteroids (Bisgrove and Burke 1986; Nakajima 1988; Moss et al. 1994; Chee and Byrne 1999; Jarvela et al. 2016). The development and localization of serotonergic neurons vary considerably between echinoderm classes, but is considered in all groups to mark the neurons of the apical organ (Byrne et al. 2007). In *A. rubens* we first observed serotonin-immunoreactive cells at gastrulation, with immunoreactivity concentrated in the lateral ganglia by 2-weeks post-fertilization. A subset of serotonin-immunoreactive cells connects the lateral ganglia across the aboral surface. As the only non-ciliary neurons on the aboral surface, these cells may also play a role in coordinating contractions of the aboral muscles that allow the bipinnaria to reverse (Strathmann 1971). As with ArPPLN1b, detailed neuroanatomical work will be required to confirm an interaction between the 2 cell types. Based on the analysis of the expression of transcripts encoding the precursor of the neuropeptide S2 (F-type SALMFamide precursor) using *in situ* hybridization (Mayorova et al. 2016), it has been suggested that this neuropeptide type is a marker of the apical organ in asteroids. Our immunohistochemical analysis of SALMFamide neuropeptide S2 indicates that it is localized in cells at the anterior end of the post-oral band but that these neurons are located slightly anteriorly to the main concentrations of serotonergic cells in the lateral ganglia (Fig. 6, right) and may represent a distinct population of cells. Further work is required to determine the function of these cells in the bipinnaria larvae.

Among studied asteroids, the extent of the serotonergic nervous system in *A. rubens* is most similar to *P. ochraceus* (Moss et al. 1994), but greatly restricted when compared with *Patiriella regularis*, where serotonin immunoreactive cells also extend into the oral region and around the oral hood (Byrne et al. 2007). Because *P. regularis* belongs to the order Valvatida while *P. ochraceus* and *A. rubens* belong to the order Forcipulatida (Supplementary Fig. S10), this may reflect a shared ancestral loss of this part of the serotonergic nervous system, or elaboration in *P. regularis* during the ∼200 million years since their evolutionary divergence.

Our work herein sheds light on the complexity of neuronal cell types and musculature present in the seemingly simple *A. rubens* larvae. This work provides the foundations for further characterization of larval development in *A. rubens* in conjunction with new genomic and transcriptomic resources for this species. Furthermore, while we have continued to expand our knowledge of asteroid development across evolutionary timescales, future work investigating the development of bipinnaria and non-bipinnaria larvae will be necessary to provide a more complete understanding of larval evolution in the Asteroidea.

## Supplementary Material

icab103_Supplementary_Data
